# Crystal Orientation Dependence of Gallium Nitride Wear

**DOI:** 10.1038/s41598-017-14234-x

**Published:** 2017-10-26

**Authors:** Guosong Zeng, Wei Sun, Renbo Song, Nelson Tansu, Brandon A. Krick

**Affiliations:** 1 Department of Mechanical Engineering and Mechanics, Lehigh University, Bethlehem, PA 18015 USA; 2 Center for Photonics and Nanoelectronics, Department of Electrical and Computer Engineering, Lehigh University, Bethlehem, PA 18015 USA

## Abstract

We explore how crystallographic order and orientation affect the tribological (friction and wear) performance of gallium nitride (GaN), through experiments and theory. Friction and wear were measured in every direction on the c-plane of GaN through rotary wear experiment. This revealed a strong crystallographic orientation dependence of the sliding properties of GaN; a 60° periodicity of wear rate and friction coefficient was observed. The origin of this periodicity is rooted in the symmetry presented in wurtzite hexagonal lattice structure of III-nitrides. The lowest wear rate was found as 0.6 × 10^−7^ mm^3^/Nm with <1$\bar{1}$00>, while the wear rate associated with <1$\bar{2}$10> had the highest wear rate of 1.4 × 10^−7^ mm^3^/Nm. On the contrary, higher friction coefficient can be observed along <1$\bar{1}$00> while lower friction coefficient always appeared along <1$\bar{2}$10>. We developed a simple molecular statics approach to understand energy barriers associated with sliding and material removal; this calculated change of free energy associated with sliding revealed that there were smaller energy barriers sliding along <1$\bar{2}$10> as compared to the <1$\bar{1}$00> direction.

## Introduction

Investigations of anisotropic tribological properties of crystal materials starting from 1960s and various crystal materials have been studied since then^1–7^. Bowden *et al*. observed that friction coefficient of MgO_2_ and diamond exhibited a 90° symmetry while with larger cone angles the friction coefficient decreased but the directionality was still preserved^2,3^. Similarly, Steijn concluded that friction and wear properties of single crystals with FCC and BCC structures, like copper, iron, NaCl, LiF, *etc*., depended on crystal orientation^4^. And for HCP crystal like sapphire, wear rate on basal plane along [$$\overline{1}$$$$\overline{1}$$20] was lower than [10$$\overline{1}$$0]^5^. After these pioneering works on anisotropic friction and wear properties, more efforts have been made in exploring directionality of tribological properties for various kind of materials^8–17^. As wurtzite single crystal material, the anisotropic properties of GaN also have been studied for decades, such as Young’s modulus, hardness, shear strength, *etc*^18–21^. On the contrary, the tribological properties (friction and wear) of GaN haven’t been systematically investigated and fully understood yet. However, knowing its wear performance is critical for understanding its processing (*e.g*. chemical mechanical polishing)^22–25^ and machinability as well as the durability of GaN-based devices exposed to harsh environments, *e.g*., space and desert, as well as in high-frequency microelectromechanical systems (MEMS). Recently, we discovered that GaN has an ultralow wear nature under dry environment and its wear property depends on the crystal orientation, with wear rate along <1$$\overline{2}$$10> being significantly higher than <1$$\overline{1}$$00>^26^. Therefore, in order to link the crystal direction to the GaN wear and map the full orientation dependence of GaN wear rate, we employed a custom rotary tribometer to conduct unidirectional (clockwise and counter-clockwise) to include all the crystal directions. A molecular static model was built to calculate energetics of an idealized wear process. This model simulated the free energy variation when dragging wear cluster around the surface.

## Results and Discussion

### Wear

A circular wear scar was obtained by a unidirectional (clockwise) rotary sliding test after 30,000 cycles under 25–30% RH lab air. The flat edge of the sapphire substrate is the a-plane (1$\overline{2}$10) and is used as reference to determine the crystal direction in the wear scar. It is important to note that the lattice of GaN has a 30° rotation from sapphire substrate in order to minimize the strain^27,28^, this means that the flat edge corresponds to the m-plane (1$\overline{1}$00) of the GaN film. This has been independently confirmed with atomic resolution transmission electron microscopy and selected area diffraction. A point of clarification for comparison: prior studies on GaN wear labeled the sliding direction based on the sapphire substrate when comparing wear of sapphire to GaN films on sapphire^26^; in the present work, we define directions based on the structure of the GaN film.

**Figure 1:**
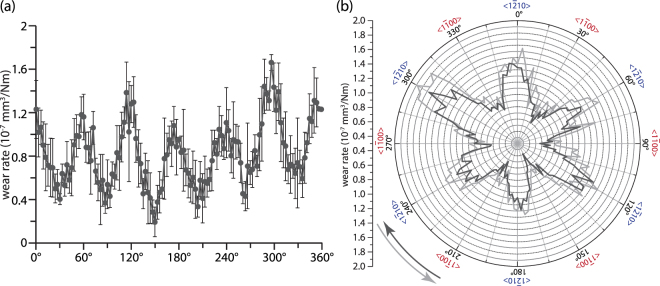
(**a**) Wear rate for clockwise unidirectional sliding test in Cartesian coordinates; (**b**) clockwise and counter-clockwise unidirectional sliding tests in polar coordinates.

GaN shows a 60° periodicity of wear rate on the c-plane (Fig. 1a), with the lowest wear rate at <1$\bar{1}$00> family direction (*K*_<1_$\bar{1}$_00>_ = 6.7 × 10^−8^ mm^3^/Nm ± 0.92 × 10^−8^ mm^3^/Nm) and the highest wear rate at <1$\bar{2}$10> family direction (*K*_<1_$\bar{2}$_10>_ = 14 × 10^−8^ mm^3^/Nm ± 2.7 × 10^−8^ mm^3^/Nm). Profilometric scans were made every 3° along this circular wear scar revealed a continuous variation of wear rate with orientation. This periodic crystallographic dependent wear behavior matches with what we reported previously that <1$\overline{1}$00> direction was more wear resistant than <1$\overline{2}$10> direction^26^. The inconsistency of the wear rate within same family direction is either attributed to the local quality of the GaN sample or a possible asymmetric within the same family direction. In order to answer this question, another unidirectional wear test with reverse sliding direction (counter-clockwise) was made on the same coating 0.5 mm away from the first unidirectional test. This is to guarantee a comparable local sample quality and allow us to differentiate the possible asymmetric wear behavior within same family direction. As plotted in Fig. 1b, we can see that the wear rate was very close to the first test with only limited increase of wear rate for all directions.

### Friction

**Figure 2:**
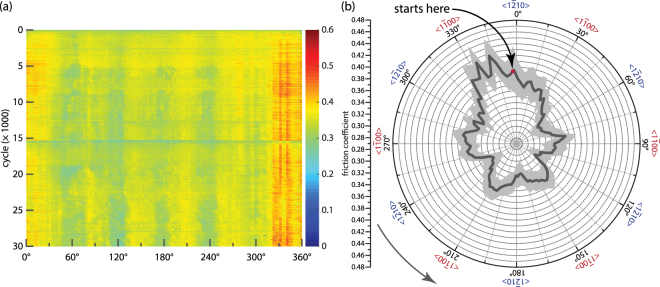
(**a**) Friction coefficient map of counter-clockwise unidirectional sliding test; (**b**) average friction coefficient of 30,000 cycles with standard deviation highlighted as light gray.

The friction coefficient of GaN also exhibited 60° periodicity, having its lowest value along <1$\overline{2}$10> and highest along <1$\overline{1}$00> (Fig. 2), except for the region around starting point of the test, which was attributed to the discontinuity of the stage near this point (acceleration and deceleration of each cycle). This periodicity does not come from variation in the normal load, which, for this experiment, typically has a ±7 mN (less than 2%) variation with periodicity of 360°, caused by slight misalignments; the friction coefficient is normalized as the instantaneous friction force divided by the instantaneous normal force. We hypothesize that a possible anisotropic energy barrier distribution is presented on the surface, which is caused by the intrinsic lattice structure of GaN.

### Molecular Statics

Unlike prior results for frictional and wear anisotropy^2,3,5^, GaN is under a very mild wear regime and plasticity is not expected to contribute. As previously reported, height removal rates for GaN are as low as ~0.0007 nm per sliding cycle^26^. To explain this, we propose a mental framework that considers the removal/sliding of a small, charge balanced GaN cluster. We hypothesize that higher energy barrier along <0$\overline{1}$00> direction results in more difficult removal process of the material, which in turn results in its lower wear rate. The anisotropy of friction coefficient is much more subtle than the anisotropy of wear rate. The anisotropy in friction likely comes from the same or similar energetic barriers derived from crystalline structure that governs wear.

**Figure 3:**
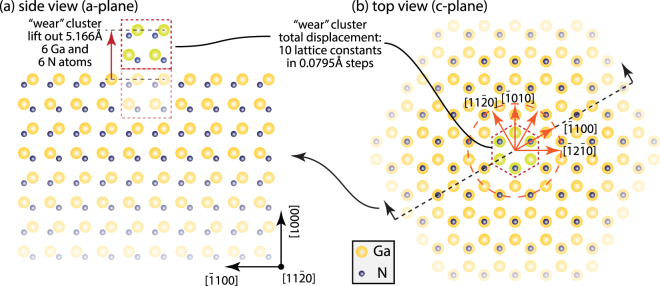
Molecular static simulation model. (**a**) Side view (a-plane) of GaN wear cluster and base (Ga-terminated), cluster lifted out of the base by 5.166 Å; (**b**) top view (c-plane), wear cluster sweeps radially at all angles along the surface with 1° increments.

To test this hypothesis, we employed molecular static calculation to understand the physics of crystal orientation dependence of wear. For simplicity, we only considered wear as the internal bond breaking within the GaN surface. Thus, only GaN-GaN interface will be simulated. Although, this model is simplified and does not strictly correspond to the complexity of the experiment, the energy barrier distribution can be used to correlate with the anisotropy of GaN’s wear performance. Simulations start with a base of GaN consisting of 54,000 Ga and 54,000 N atoms. A small cluster of 6 Ga atoms and 6 N atoms was extracted from the center of the top surface of the base and moved up 5.166 Å (Fig. 3a), at a location in registry with the GaN crystal. The reference total energy of the system was first determined when the wear cluster was placed right above the hole it left behind at a distance in registry with the base. This total energy was obtained by summing all energy potentials between each cluster atom and every atom in the base (sum of 1,295,856 potentials for each iteration). The twelve-atom “wear” cluster was then “slid” radially from the center in 0.0795 Å incremental steps out to 31.8 Å (400 steps for 10 lattice spacings from the origin). The radial sweeps started at [$\overline{1}$2$\overline{1}$0] and were done at all angles along the surface, in 1 degree increments. The total energy for each location is calculated. A two-body potential was used to obtain the interaction between Ga-Ga, Ga-N and N-N^29^. The variation of total energy associating with cluster in different places along different crystal direction then was revealed.

**Figure 4:**
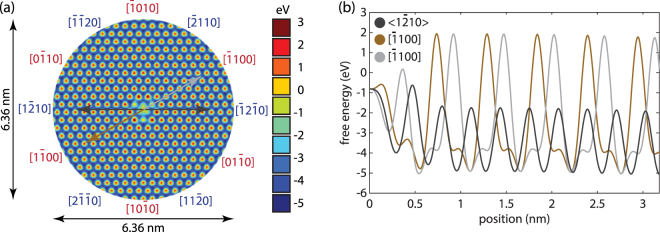
(**a**) Free energy variation of GaN wear cluster moving along the GaN surface; (**b**) line scans of the energy barriers along [$\overline{1}$010], [$\overline{1}$100]; [$\overline{2}$110] and [$\overline{1}$2$\overline{1}$0] overlapped and plotted as family direction <1$\overline{2}$10>.

The molecular static simulation results revealed 60° periodicity on c-plane surface, with energetic barriers to sliding strongly linked to the sliding direction. This supports our hypothesis that the crystallographic orientation dependences of wear rate is attributed to the anisotropic energy barrier distribution on the c-plane surface of GaN. From Fig. 4, we can see that there are less energy barriers along <1$\bar{2}$10> family direction while more energy barriers along <1$\overline{1}$00> family direction. This indicates that more work is required to overcome the energy barriers along the <1$\overline{1}$00> direction, resulting in lower wear rate. On the contrary, less energy barriers make the wear cluster more free to pass along the <1$\overline{2}$10> direction, leading to increased wear rate in this direction (Fig. 4a). We also plot the energy variations along specific crystallographic directions (Fig. 4b). Higher energy barrier height in <1$\overline{1}$00> than in <1$\overline{2}$10> means it is more difficult to drag a wear cluster along <1$\overline{1}$00> family direction, again demonstrating that <1$\overline{1}$00> family direction has a lower wear rate than <1$\overline{2}$10>. It is also noteworthy that the crystal structure along <1$\overline{2}$10> family direction strictly repeats every 60° while the crystal structure in <1$\overline{1}$00> is exact the same every 120°, but varies a little every 60° (Fig. 4b). To specify this point, we looked into the crystal structure of GaN (Fig. 3) and saw that when moving along [1$\overline{1}$00] direction, the wear cluster will approach one Ga atom first and split two other N atoms afterwards. On the contrary, when moving along [$\overline{1}$100] (the opposite direction) the wear cluster will split two N atoms first then approach the next Ga atom. This slight difference is due to wurtzite layers cycling among two equivalent shifted layers; we suspect that there is no anisotropy in wear between these two directions, as this effect is averaged out as the material is worn through multiple layers. Theoretically speaking, this anisotropy in <1$\overline{1}$00> family direction could exist instantaneously and in atomic-scale wear events and experiments. However, it is important to note that the wear behavior of GaN is influenced not only by crystal orientation, but also highly depends on the humidity, as well as the local sample quality, which makes it extremely difficult to demonstrate this theoretical calculation with macroscale wear test.

## Conclusions

To conclude, we explored the full map of crystallographic orientation dependence of GaN’s wear behavior by means of both experimental and computational methods. This demonstration of the crystallographic orientation wear dependence of GaN provides the further understanding beyond our initial discovery on the low wear rate of this material. From the experimental results, we can observe that wear performance of GaN exhibits a strong directionality with a periodicity of 60°, with local lowest always appeared in <1$\overline{1}$00> family direction and local highest wear rate in <1$\overline{2}$10> family direction. Molecular static simulation helps us to explain the physics behind crystallographic dependence. Higher energy barrier density and height along <1$\overline{1}$00> family direction than in <1$\overline{2}$10> require more work to move around the wear cluster, which in turn explains the reason why <1$\overline{2}$10> family direction has higher wear rate than <1$\overline{1}$00> family direction. Consequently, the current finding provides further insight into crystallographic orientation dependence of wear behavior of GaN and benefits the GaN society during device designs and applications.

## Materials and Methods

**Figure 5:**
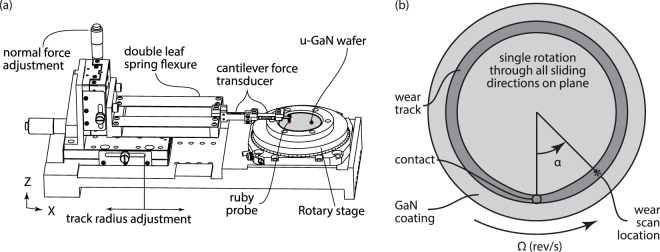
(**a**) Schematic of rotary tribometer; (**b**) illustration of the unidirectional wear experiment and profilometric scan.

Unintentionally doped single crystal (0001)-plane GaN coating (3 µm thick, grown by metal organic chemical vapor deposition on c-plane single crystalline sapphire) was used in these experiments. The as-grown u-GaN sample has background doping (n-type) of n ~ 5×10^16^ cm^−3^. The rotary wear tests were performed by our custom rotary tribometer (Fig. 5a) mounted inside a glovebox to achieve the environment control (25–30% RH lab air). Two force transducers attached to the flexure were used to measure the normal and frictional forces. The linear sliding speed was set to be 20 mm/s and the sliding cycles was 30,000. A single crystal ruby ball (α-alumina) with radius of 1.5 mm (Swiss Jewel Company, Grade 25) was used as the countersample due to its hardness and wear resistance. The applied normal load was set to be 600mN (~1.2 GPa maximum Hertzian contact pressure, approximately 1/10 of the hardness of GaN^30–34^). The rotary wear test was first performed in a unidirectional (clockwise) manner for obtaining the wear rate corresponding to each crystal direction (Fig. 5b), then a second unidirectional rotary was conducted with reversal direction, in order to distinguish the symmetry of wear behavior of wurtzite GaN. Optical profilometer (Bruker ContourGT-K) was used to measure the cross-sectional area of the wear scar. The rotary stage carrying the sample was placed underneath the profilometer for wear scar profile measurement (Fig. 5b). The stage rotation step of 3° was used to achieve a full profilometric scan. Then Archard wear rate was used for deriving the wear rate^35^. Five line scans within each single profilometric scan were made to get data that is statistically representative of the local material wear properties^36^.

## References

[1] Buckley DH, Miyoshi K (1984). Friction and wear of ceramics. Wear.

[2] Bowden FP, Brookes CA, Hanwell AE (1964). Anisotropy of Friction in Crystals. Nature.

[3] Bowden FP, Brookes CA (1966). Frictional anisotropy in nonmetallic crystals. Proc. R. Soc. A Math. Phys. Eng. Sci..

[4] Steijn RP (1964). Friction and Wear of Single Crystals. Wear.

[5] Steijn RP (1961). On the wear of sapphire. J. Appl. Phys..

[6] Sawyer WG, Argibay N, Burris DL, Krick BA (2014). Mechanistic Studies in Friction and Wear of Bulk Materials. Annu. Rev. Mater. Res..

[7] Duwell EJ (1962). Friction and wear of single-crystal sapphire sliding on steel. J. Appl. Phys..

[8] El-Hadad S, Sato H, Watanabe Y (2012). Investigation of wear anisotropy in a severely deformed Al–Al3Ti composite. Metall. Mater. Trans. A Phys. Metall. Mater. Sci..

[9] Gatzen HH, Beck M (2003). Investigations on the friction force anisotropy of the silicon lattice. Wear.

[10] Kadijk SE, Groenou ABVAN (1990). Wear anisotropy of MnZn ferrite part I: recorder and sphere-on-tape experiments. Wear.

[11] Liley M (1998). Friction Anisotropy and Asymmetry of a Compliant Monolayer Induced by a Small Molecular Tilt. Science (80-.)..

[12] Miyoshi K, Buckley DH (1982). Anisotropic tribological properties of SiC. Wear.

[13] Namai Y, Shindo H (2000). Frictional force microscopic anisotropy on (001) surfaces of alkali halides and MgO. Jpn. J. Appl. Phys..

[14] Park JY (2005). High frictional anisotropy of periodic and aperiodic directions on a quasicrystal surface. Science.

[15] Pastewka L, Moser S, Gumbsch P, Moseler M (2011). Anisotropic mechanical amorphization drives wear in diamond. Nat. Mater..

[16] Stempfle P, Takadoum J (2012). Multi-asperity nanotribological behavior of single-crystal silicon: Crystallography-induced anisotropy in friction and wear. Tribol. Int..

[17] Yu B, Qian L (2013). Effect of crystal plane orientation on the friction-induced nanofabrication on monocrystalline silicon. Nanoscale Res. Lett..

[18] Fujikane, M., Yokogawa, T., Nagao, S. & Nowak, R. Nanoindentation study on insight of plasticity related to dislocation density and crystal orientation in GaN. *Appl. Phys. Lett*. **101** (2012).

[19] Kisielowski C (1996). Strain-related phenomena in GaN thin films. Phys. Rev. B.

[20] Moram MA, Vickers ME (2009). X-ray diffraction of III-nitrides. Reports Prog. Phys..

[21] Savastenko VA, Sheleg AU (1978). Study of the elastic properties of gallium nitride. Phys. Status Solidi.

[22] Tavernier PR, Margalith T, Coldren LA, DenBaars SP, Clarke DR (2002). Chemical Mechanical Polishing of Gallium Nitride. Electrochem. Solid-State Lett..

[23] Weyher JL, Müller S, Grzegory I, Porowski S (1997). Chemical polishing of bulk and epitaxial GaN. J. Cryst. Growth.

[24] Aida H (2014). Surface Planarization of GaN-on-Sapphire Template by Chemical Mechanical Polishing for Subsequent GaN Homoepitaxy. ECS J. Solid State Sci. Technol..

[25] Hayashi S, Koga T, Goorsky MS (2008). Chemical Mechanical Polishing of GaN. J. Electrochem. Soc..

[26] Zeng G, Tan CK, Tansu N, Krick BA (2016). Ultralow wear gallium nitride. Appl. Phys. Lett..

[27] Akasaka T, Kobayashi Y, Ando S, Kobayashi N (1997). GaN hexagonal microprisms with smooth vertical facets fabricated by selective metalorganic vapor phase epitaxy. Appl. Phys. Lett..

[28] Grandjean N, Massies J, Leroux M (1996). Nitridation of sapphire. Effect on the optical properties of GaN epitaxial overlayers. Appl. Phys. Lett..

[29] Harafuji K, Tsuchiya T, Kawamura K (2004). Molecular dynamics simulation for evaluating melting point of wurtzite-type GaN crystal. J. Appl. Phys..

[30] Yang Z (2006). Mechanical characterization of suspended GaN microstructures fabricated by GaN-on-patterned-silicon technique. Appl. Phys. Lett..

[31] Tsai CH, Jian SR, Juang JY (2008). Berkovich nanoindentation and deformation mechanisms in GaN thin films. Appl. Surf. Sci..

[32] Nowak R (1999). Elastic and plastic properties of GaN determined by nano-indentation of bulk crystal. Appl. Phys. Lett..

[33] Drory MD, Ager JW, Suski T, Grzegory I, Porowski S (1996). Hardness and fracture toughness of bulk single crystal gallium nitride. Appl. Phys. Lett..

[34] Kucheyev SO (2000). Nanoindentation of epitaxial GaN films. Appl. Phys. Lett..

[35] Erickson GM (2016). Paleo-tribology: development of wear measurement techniques and a three-dimensional model revealing how grinding dentitions self-wear to enable functionality. Surf. Topogr. Metrol. Prop..

[36] Colbert RS (2011). Uncertainty in Pin-on-Disk Wear Volume Measurements Using Surface Scanning Techniques. Tribol. Lett..

