# Publisher Correction: Crystal Orientation Dependence of Gallium Nitride Wear

**DOI:** 10.1038/s41598-018-19513-9

**Published:** 2018-02-02

**Authors:** Guosong Zeng, Wei Sun, Renbo Song, Nelson Tansu, Brandon A. Krick

**Affiliations:** 1 Department of Mechanical Engineering and Mechanics, Lehigh University, Bethlehem, PA 18015 USA; 2 Center for Photonics and Nanoelectronics, Department of Electrical and Computer Engineering, Lehigh University, Bethlehem, PA 18015 USA

Correction to: *Scientific Reports* 10.1038/s41598-017-14234-x, published online 26 October 2017

The original PDF version of this Article contained missing labels in Figure 3. This has now been corrected in the PDF; the HTML version of the paper was correct from the time of publication.

